# Disinfection and Biocompatibility of Titanium Surfaces Treated with Glycine Powder Airflow and Triple Antibiotic Mixture: An In Vitro Study

**DOI:** 10.3390/ma15144850

**Published:** 2022-07-12

**Authors:** Mario Alovisi, Massimo Carossa, Narcisa Mandras, Janira Roana, Massimo Costalonga, Lorenza Cavallo, Enrico Pira, Maria Grazia Putzu, Davide Bosio, Ilaria Roato, Federico Mussano, Nicola Scotti

**Affiliations:** 1 Department of Surgical Sciences, CIR Dental School, University of Turin, Via Nizza 230, 10126 Turin, Italy; mario.alovisi@unito.it (M.A.); ilaria.roato@unito.it (I.R.); federico.mussano@unito.it (F.M.); nicola.scotti@unito.it (N.S.); 2 Department of Public Health and Pediatrics, University of Turin, 10126 Turin, Italy; narcisa.mandras@unito.it (N.M.); janira.roana@unito.it (J.R.); lorenza.cavallo@unito.it (L.C.); enrico.pira@unito.it (E.P.); 3 Diagnostic and Biological Sciences, University of Minnesota, Minneapolis, MN 55455, USA; costa002@umn.edu; 4 Unit of Occupational Medicine and Hospital Occupational Unit, A.O.U Città della Salute e della Scienza di Torino, 10126 Turin, Italy; mariagrazia.putzu@unito.it (M.G.P.); davide.bosio@unito.it (D.B.)

**Keywords:** peri-implantitis, airflow, antibiotics, confocal laser scanning microscopy, adipose tissue-derived mesenchymal stem cells, implant-supported dental prostheses, biocompatibility testing

## Abstract

The aim of this in vitro study was to compare three disinfection protocols of biofilm-coated machined (MAC) and acid etched (SLA) commercial pure Grade 4 Titanium disks. Samples were infected with a vial of polymicrobial biofilm to simulate peri-implantitis in vitro. Seventeen MAC and twenty SLA titanium disks were randomly assigned to: (1) glycine powder air-flow (GYPAP) for 1 min; (2) a local delivered triple paste antibiotic composed by a gel mixture with ciprofloxacin, metronidazole, and clarithromycin (3MIX) for 1 h; and (3) a combination of both (GYPAP + 3MIX). Biocompatibility of the titanium disks after each treatment protocol was assessed by measurement of adhesion and growth of adipose-derived mesenchymal stem cells (ASCs) after 24 and 72 h. A confocal laser scanning microscope (CLSM) assessed the antibacterial effect of each treatment. Data of the antibacterial efficacy and cell viability were presented as mean with standard deviation and calculated by one-way ANOVA with multiple comparisons via Bonferroni tests. Results were considered significant with *p* < 0.05. The higher cell viability was achieved by the 3MIX and GYPAP combination on the SLA surfaces after 72 h. CLSM analysis showed a mean ratio of dead bacteria statistically higher in the 3MIX + GYPAP group compared with the GYPAP and 3MIX subgroups (*p* < 0.05). In conclusion, data showed that the combination of GYPAP and 3MIX could be preferred to the other protocols, especially in presence of SLA titanium surface.

## 1. Introduction

Dental implants have achieved a predominant role in daily clinical practice and are currently considered the first instrument of choice to rehabilitate the edentulous areas [1]. Failures, however, remain an unfortunate clinical event that is yet to be addressed. These failures are usually classified as early or late. Early failures may be caused by surgical trauma, low quality or quantity of bone, inadequate primary stability, and bacterial infection of the recipient site and are attested around 2% of the cases [2,3]. Late failures are associated either with masticatory overload or peri-implantitis and range among 5–10% [3,4].

Peri-implantitis (PI), also known as implant infection, is a biological complication involving soft and hard tissues around the osseointegrated implants [5,6]. It consists of a polymicrobial anaerobic infection which causes a chronic inflammatory process [5,7], leading to the formation of a peri-implant pocket with consequent bone loss [8,9]. PI prevalence has been recently estimated to be involved in more than 12% of the implant treatments in the clinical practice [10,11]. The decontamination of the implant surface and the consequent resolution of the tissues’ inflammation represent the main goal that should be achieved in the treatment of PI. During the last decade, different methods have been reported to minimize or remove biofilms from contaminated surfaces [12]. Chemical and air-abrasive treatments appear to be capable of disrupting the bacterial biofilm [13]. Conversely, chemical cleaning solutions in combination with mechanical debridement may be ineffective [14]. Inconsistent results were presented by lasers or photodynamic therapy [15,16]. Interestingly, implantoplasty remains somehow a preferred way to remove infected contaminants during the clinical practice [17]. When the reosseointegration of the treated implants is assessed, the quality of the decontaminated implant surfaces is deemed an important predictor of the future outcome. Therefore, some studies have questioned the real effectiveness of implantoplasty as a treatment of PI [18]. Other authors pointed out how implantoplasty leads to an increase in stress at the implant level and to a tissue dispersion of biofilm-contaminated titanium particles and, consequently, they discouraged this treatment option [19]. Nevertheless, different local delivered antiseptics and antibiotics have been tested to lower or eliminate the local bacteria load [16,20]. Despite some positive effects, the complete resolution of the pathology is usually not complete. Then, the treatment of the PI is still challenging, and a final protocol remains a matter of discussion.

Additionally, surface characteristics are usually strictly related to clinical behavior and host response to different materials [21]. Concerning implant surfaces, different surface topographies were proposed over the years to increase cell adhesions [22,23]. Among others, machined (MAC) and acid etched (SLA) surfaces are some of the most common surfaces that were analyzed over the years. As described in detail in the review by Wennerberg and Albrektsson [22], MAC surfaces represent a smooth surface obtained through a manufacturing process that can be turned, polished, or milled. On the other hand, SLA surfaces indicate a rough surface, which is obtained by removing a small amount of surface material through an etching process.

The first study hypothesis of the present in vitro study was to compare the effect of different decontamination protocols on the disinfection of contaminated titanium disks (MAC and SLA Ti surfaces). The second study hypothesis was to assess the biocompatibility of the treated titanium disks with adhesion and grow of adipose-derived mesenchymal stem cells (ASCs).

The null hypothesis was that different protocols can influence the disinfection and the consequent biocompatibility of infected titanium disks.

## 2. Materials and Methods

### 2.1. Saliva Collection

Saliva samples were collected and pooled (approx. 50 mL/donor) from ten adult donors that voluntarily underwent the withdrawal. Informed consent was obtained from all subjects involved in the study and the local ethical committee approved the study protocol (Approval code: DS_000161_2022). The inclusion criteria considered healthy participants with normal salivary flow and absence of active caries or periodontal disease. To ensure standardization, patients refrained from oral hygiene measures overnight.

The collected unstimulated saliva samples were diluted in sterile saline at 1:10 dilution and filtered through filter paper (Wharman, Merk, Germany) to remove large debris. The prepared saliva was stored in aliquots of 50 mL and frozen at −80 °C for further use [24].

### 2.2. Biofilm Formation on Disks

**Figure 1:**
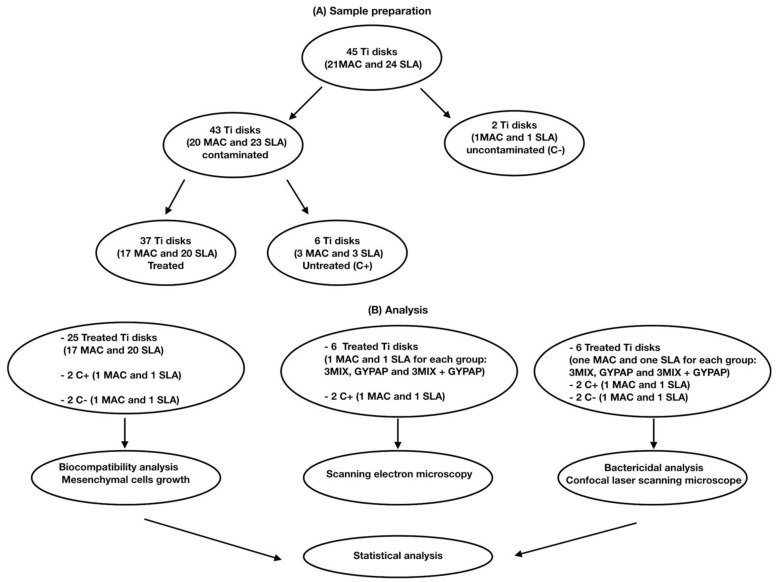
Flowchart of the study. (**A**) Sample preparation. (**B**) Analysis.

Flowchart of the study is shown in Figure 1.

Twenty-one MAC Titanium disks and twenty-four SLA Titanium disks were utilized after sterilization procedures. All disks were made of commercial pure Grade 4 titanium. A sample size of 20 was calculated with G*Power 3.1.4 (Kiel University, Kiel, Germany) to set the study power at 80% considering an alpha error = 0.05. 

Two disks (1 MAC and 1 SLA) were left uncontaminated (C−), while a polymicrobial biofilm was grown on the remaining titanium disks (43 disks). A total of 37 disks were randomly assigned to 3 different groups by means of a computer-generated sequence (SPSS 24.0; SPSS Inc., Chicago, IL, USA), while 6 disks were used to assess biofilm formation (C+). To develop a vial polymicrobial biofilm in vitro, the following bacteria were isolated from plaque samples and used: *Capnocytophaga ochracea* and *Streptococcus salivarius 3b*. The identification of bacteria was performed by mass spectrometry (MALDI-TOF, Bruker Labscape, Bruker Belgium S.A./N.V, Kontich, Belgium). 

*Enterococcus faecalis* ATCC 29212, *Fusobacterium nucleatum* ATCC 25586, *Staphylococcus epidermidis* ATCC 35984, and *Streptococcus mutans* ATCC 35668 were also included. The six multispecies biofilms used to contaminate the titanium disks are the essential bacterial strains present in PI sites. Biofilm was grown on disks in a medium consisting of 60% whole unstimulated saliva and 40% Tryptic Soy Broth (TSB). 

Briefly, each bacterial strain was separately cultured on CDC ANAEROBE +5% SB plates for 48 h at 37 °C in CO_2_ (*C. ochracea*, *E. faecalis*, *S. mutans,* and *S. salivarius*) or in aerobic (*S. epidermidis*)/anaerobic conditions (*F. nucleatum*). Then, a 4 McFarland bacterial suspension (1.2 × 10^9^ CFU/mL) in TSB was prepared and the treatment was performed over the following six days.

In details: 43 disks were incubated in defrosted, pooled saliva in anaerobic/microaerophilic conditions at room temperature for 4 h to promote the formation of the acquired pellicle [25]. Next, saliva was substituted with defrosted, pooled saliva, TSB, and mixed bacterial suspension (to a density of 4 McFarland per each strain [1.2 × 10^9^ CFU/mL]). Afterwards, a new bacterial suspension was added. The medium TSB was replaced with a fresh medium plus saliva. They were then incubated at 37 °C in CO_2_ to form a mature biofilm and planktonic cells were removed under sterile conditions [26,27]. Finally, the medium TSB was replaced with fresh medium plus saliva and incubated at 37 °C for 24 h in CO_2_.

### 2.3. Treatment Procedures

Six disks (three MAC and three SLA) contaminated with the polymicrobial biofilm were untreated and utilized as positive controls. The remaining 37 contaminated disks were randomly assigned to 3 different test groups through a computer-generated random sequence of numbers (SPSS 24.0; SPSS Inc., Chicago, IL, USA), as previously described [28,29]. Two groups were composed of 6 MAC and 6 SLA disks each, and one group was composed of 6 MAC and 7 SLA disks. The decontamination of the disks was performed according to the following procedures:

1. The application of an antibiotic gel mixture with ciprofloxacin, metronidazole, and clarithromycin (3MIX) for 1 h.
2. Glycine powder air polishing (GYPAP) for 1 min (AirFlow Prophylaxis Master, EMS Dental) set at medium intensity.
3. The combination of the two previous procedures (3MIX + GYPAP), first the application of the antibiotic gel mixture and after glycine powder air polishing.

### 2.4. Mesenchymal Cells Growth on Treated Disks

ASC52telo (ASCs), hTERT immortalized adipose-derived mesenchymal stem cells were cultured according to ATCC protocols. Briefly, cells were expanded in Mesenchymal Stem Cell Basal Medium (ATCC PCS-500-030) with Mesenchymal Stem Cell Growth Kit (ATCC PCS-500-040) (ATCC, Manassas, VA, USA). Immediately after decontamination procedures, 1 × 10^5^ cells/mL ASCs were seeded onto the top of 25 disks plus 2 positive controls (saliva coating and bacterial biofilm) and 2 negative controls (saliva coating). Cells were cultured in alpha-MEM (Lonza) and supplemented with 10% Fetal Bovine Serum (FBS) at 37 °C in a humidified atmosphere with 5% CO_2_ for 3 days. 

To assess the effect of each decontamination protocol on the ASCs’ viability, Neutral Red viability assay (Sigma-Aldrich, St. Louis, MO, USA) was performed according to the manufacturer’s instructions to monitor the growth of the cells after 24 and 72 h of culture.

### 2.5. Influence of Decontamination on ASCs’ Adhesion

To assess the effect of each decontamination protocol on the ASCs’ adhesion to the sample surface, the cells that were incubated for at least 24 h were fixed with 2.5% glutaraldehyde in buffered saline solution, dehydrated through a graded series of ethanol, dried, and observed for morphological assessments and Backscattered Electron Microscope (BES) for semiquantitative analysis (Jeol Nikon JCM-6000P, at 15 kV). The specimens were photographed by a blind operator. For each disk, (a) one photo at low magnification (20×) was taken to describe cells distribution on disk surface; (b) 6 photos at a total magnification of 55× were taken in the area where cells had been seeded to perform semiquantitative analysis; and (c) high-magnification photos (440× to 1500×) were taken on a few randomly selected samples to assess the cellular morphology. The percentage of disks’ surface covered by adherent cells was calculated by the same blind operator (IR) on 55× photos using an image analysis system (ImageJ, NIH, Bethesda, MD, USA). 

### 2.6. Scanning Electron Microscopy

Six Ti disks were analyzed with scanning electron microscopy (SEM) (one MAC and one SLA for each group: 3MIX, GYPAP, and 3MIX + GYPAP) to visualize the presence of biofilm on the surface after 3 different decontamination treatments. Two positive controls were analyzed for each MAC and SLA group. Specimens were fixed in 2.5% glutaraldehyde overnight at 4 °C and then dehydrated through a graded sequence of ethanol at different concentrations. MAC and SLA disks were examined using SEM (Emission Scanning Electron Microscopy, Zeiss Supra 40 Field) with different magnifications.

### 2.7. Confocal Laser Scanning Microscope (CLSM) Analysis

A live/dead BacLight bacterial viability kit (Molecular Probes Inc. Eugene, OR, USA) containing two fluorescent dyes propidium iodine (PrI) and SYTO9 was used to determine the viability of the bacteria after the different treatments.

Before examination, 6 specimens (one MAC and one SLA for each group: 3MIX, GYPAP, and 3MIX + GYPAP) were stained with fluorescent dye and incubated in the dark at room temperature for 30 min. Then, samples were rinsed with PBS to remove the extra dye and were covered with mounting oil. Each disk was analyzed by confocal laser scanning microscopy (Leica SP8, Leica Microsystems, Wetzlar, Germany). Two negative controls and two positive controls were analyzed for each MAC and SLA group.

Imaging was performed using a Leica SP8 confocal system (Leica Microsystems, Wetzlar, Germany) [30] equipped with an argon ion and a 561 nm DPSS lasers. Specimens were mounted on an inverted microscope illuminated by a krypton/argon laser (488 nm). Emission wavelengths of 505–550 nm (green, SYTO 9) and 650–750 nm (red, PrI) were utilized to visualize SYTO 9 (live bacteria) and PrI (dead bacteria), respectively. Samples were imaged using a HCX PL APO 20×/0.75 NA objective. A series of x-y-z images were collected. Laser power and detector gain were set on the control sample and kept identical for all conditions of the experiment. Images were then analyzed with ImageJ software (NIH, Bethesda, MD, USA). 

Bacterial viability (%) was calculated from the CLSM images. The central portion of each disk was analyzed through the visualization of a grid and a blind examiner took the pictures for each group. For the tested samples, the mean decontamination procedure antibacterial efficacy was calculated from 10 separate measurements for each single image, adjusted for the red color channel. The ratio of red fluorescence to green-and-red fluorescence (indicating the proportion of dead bacteria for each group) was calculated from merged images and three-dimensional reconstructions. 

### 2.8. Statistical Analysis

All statistical analyses were carried out using GraphPad Prism 6 (GraphPad Software, San Diego, CA, USA). Data were presented as mean with standard deviation and calculated by one-way ANOVA with multiple comparisons via Bonferroni tests. Results were considered significant with *p* < 0.05. 

## 3. Results

### 3.1. Effect of Different Decontamination Protocols on Bacterial Viability

**Figure 2:**
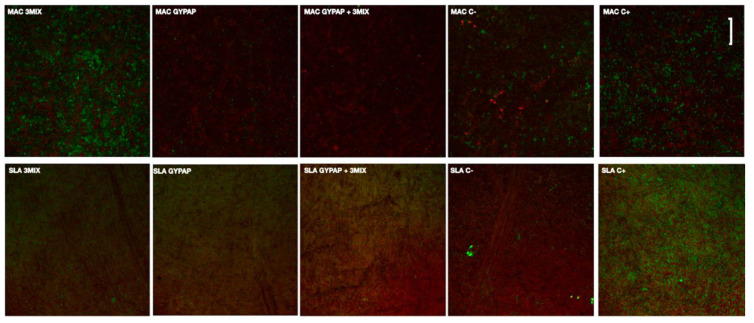
Confocal laser scanning microscopy (CLSM) images of the machined (MAC) and acid etched surface (SLA) titanium surfaces with two different decontamination protocols. The scale bar was set to 10 microns.

Viable and dead bacteria after each treatment are shown in CLSM images (Figure 2).

A biofilm layer with a prevalence of viable cells (green fluorescence) was formed on positive controls of tested surfaces. A strong decrease and viability of bacteria were observed for the SLA_ 3MIX + GYPAP and MAC groups. 

**Figure 3:**
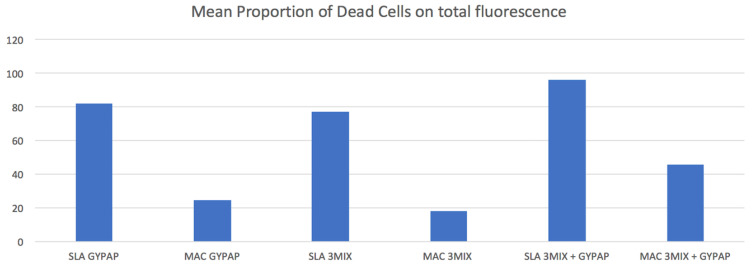
Proportion of dead cell volume of the machined (MAC) and acid etched surface (SLA) titanium surfaces treated with two different decontamination protocols. X-axis indicates the treatment’s protocol groups. Y-axis indicates the mean proportion (%) of dead cells.

Red fluorescence ratios for each group are reported in Figure 3.

In the SLA and MAC groups, the mean ratios of red fluorescence were statistically higher in the 3MIX + GYPAP subgroups compared with the GYPAP and the 3MIX subgroups (*p* < 0.05). SLA_3MIX + GYPAP surfaces showed a bactericidal efficacy almost twice that of MAC_3MIX + GYPAP (*p* < 0.05). 

### 3.2. SEM Analysis

**Figure 4:**
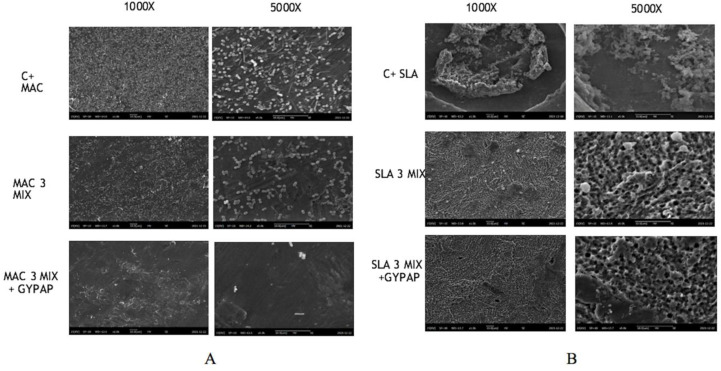
Scanning electron microscopy (SEM) images of the machined (MAC) and acid etched surface (SLA) titanium surfaces at magnifications of 1000× and 5000× with two different decontamination protocols. From left to right: (**A**) MAC, (**B**) SLA. From top to bottom: (i) positive control, (ii) after 3MIX treatment, (iii) after 3MIX + GYPAP treatment.

Figure 4 shows SEM images of the machined (MAC) and acid etched surface (SLA) titanium disks at magnifications of 1000× and 5000×, both before and after different decontamination protocols.

Generally, biofilm appeared to be denser and more firmly attached to SLA titanium surface than on MAC titanium surface before and after treatments. MAC and SLA positive controls consist of a complex multilayered structure in which micro-organisms are involved in a thick matrix. The surface disappears under the mature biofilm structure but mixed bacterial species, such as cocci and bacilli, can be especially observed in SLA. 

The 3MIX + GYPAP disinfection protocol appeared more efficient in removing bacteria from the MAC and SLA surfaces compared with the other groups. 

After treatments, SEM analyses revealed distinct alterations of the adherent micro-organisms and the biofilm matrix on the biofilm-covered MAC titanium disks: a significant reduction was recorded for MAC_3MIX + GYPAP, where the presence of cocci and bacilli was scattered on the surfaces of disks.

### 3.3. Effects of Different Decontamination Protocols on ASC Growth

**Figure 5:**
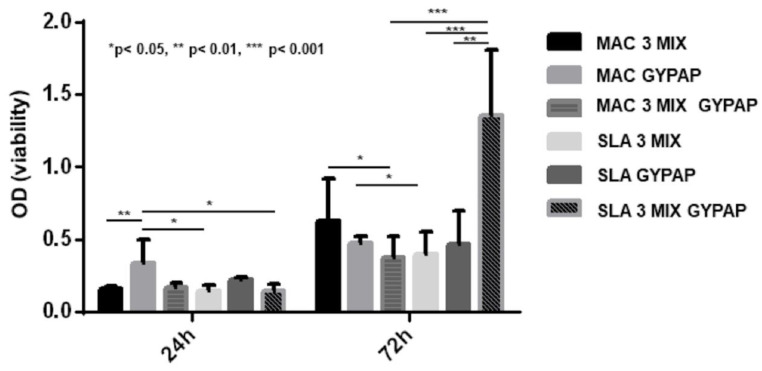
ASC viability. The graph shows the ASC viability on the disks decontaminated, after 24 and 72 h.

All the decontamination techniques appeared effective since ASCs adhered and grew during 72 h (Figure 5). After 24 h of culture, the decontaminated surface which allowed ASC growth was MAC_GYPAP, showing an increased cell viability compared to SLA_3 MIX, SLA_3 MIX + GYPAP (*p* < 0.05), and MAC_3 MIX (*p* < 0.01), SLA_3MIX (*p* > 0.05), MAC_3MIX + GYPAP (*p* < 0.01), and SLA_ 3MIX + GYPAP (*p* < 0.01). After 72 h, ASCs continued to grow on all the decontaminated surfaces; in particular, the growth was significantly higher for MAC_3MIX vs. MAC_3 MIX + GYPAP (*p* < 0.05). The best decontaminated surface growth appeared for SLA_3MIX + GYPAP compared to SLA_GYPAP (*p* < 0.01), SLA_3MIX (*p* < 0.001), and MAC_3MIX + GYPAP (*p* < 0.001).

## 4. Discussion

A vital polymicrobial biofilm including six essential bacterial strains (*Capnocytophaga ochracea, Streptococcus salivarius 3b, Enterococcus faecalis, Fusobacterium nucleatum, Staphylococcus epidermidis,* and *Streptococcus mutans*) was prepared to simulate, in vitro, a PI. Three different decontamination protocols of planar titanium surfaces were compared: (a) a mechanical treatment based on glycine air-flow (GYPAP), (b) a local-delivered triple paste antibiotic (3MIX), and (c) a combination of both (3MIX + GYPAP). Following a previous study [31], ASCs were selected as mesenchymal precursors of osteoblasts and were seeded and grown on the titanium disks after decontamination to perform a cell viability assay (Figure 2). Different decontamination protocols produced different outcomes in terms of disinfection and biocompatibility of the infected titanium disks and the null hypothesis was accepted. The higher proliferation of ASCs on SLA than on MAC is in accordance with the literature, where the surface topography is considered a key factor for cell adhesion, and rough surfaces are preferred to smooth ones [22]. The capability of GYPAP to disrupt and eliminate the biofilm from the surface was assessed, after only 24 h of culture, by the highest cell growth compared to other treatments. Notably, indeed, at the end of the third day of culture, the efficacy of the cleansing techniques varied according to the type of surface investigated. The triple paste antibiotic 3MIX outperformed even its combination with GYPAP on the smooth surface (MAC), whilst the best viability data were achieved by the 3MIX and GYPAP combination on the rough surface (SLA). GYPAP is routinely used for implant professional oral hygiene [32,33] and for the nonsurgical treatment of peri-implant diseases [34]. A recent systematic review by Schwarz et al. [34] concluded that GYPAP is an effective procedure for the treatment of mucositis, while its usage was described to be controversial for the treatment of PI [34]. Although positive effects in the reduction of some disease indicators, such as bleeding upon probing, were attained, long-term, positive outcomes seemed inconclusive.

These inconsistencies may depend also on the type of surface investigated, as it is obvious that the rougher the surface, the more difficult the mechanical removal of a given bacterial biofilm is. The SEM images portrayed in Figure 2 showed differences between biofilm formation on SLA and MAC as regards the total amount of bacteria. This difference was further enhanced after the mechanical cleansing where the SLA disks harbored a complex and firmly attached biofilm while the MAC disks hosted a biofilm that presented with a pattern of spread bacteria forming fewer clusters.

Some authors hypothesized that, despite their capability in removing the biofilm, mechanical nonsurgical treatments of PI may not be able to remove bacteria products, such as lipopolysaccharide (LPS) [35,36,37]. These bacterial products have been demonstrated to be responsible for negative interactions with the cells’ attachment, which are mandatory in order to obtain bone regeneration. Bor-Shiunn Lee et al. [35] investigated the ability of different mechanical nonsurgical treatments in the elimination of the LPS, highlighting how mechanical nonsurgical treatments are not sufficiently effective in eliminating the LPS and increasing the cell adhesion. Other authors reported how impaired access around the prosthetic reconstruction [38,39] and surface roughness of the contaminated implant [40,41] limit the reduction of the bacterial load at sites with PI, and how resolution of inflammation is often incomplete [42].

To address this pitfall, different, local-delivered antiseptics and antibiotics in the treatment of PI were proposed over the years. Mombelli et al. [43] found positive effects by local delivery of tetracycline on the treatment of initial PI. Renvert et al. [44,45] investigated the effects of chlorexidina gel. Bassetti et al. [46] used minocycline in the treatment of PI. However, complete resolution of PI was not routinely achieved in any cases and the treatment of PI is still a matter of discussion. Given these premises, the use of topical triple paste was proposed in this study, owing to the success in the regeneration and revascularization of immature teeth with apical periodontitis [47,48]. Recently, the mixture of ciprofloxacin, metronidazole, and clarithromycin (3MIX) has been suggested as a viable triple antibiotic paste because of the presence of clarithromycin. This advanced generation of macrolid antibiotics exhibits excellent activity on a large spectrum of bacteria and optimum anti-inflammatory properties, both intra- and extracellular [49,50]. Nevertheless, it provides anti-inflammatory activities with minimum side effects [51,52].

The combination of GYPAP and 3MIX could increase biocompatibility in the rough surface, assuming even more interest with the CLSM analysis (Figure 2). Here, mechanical and chemical cleansing together did not result in the complete elimination of the biofilm, but a remarkable reduction (96%) of the number and viability of planktonic bacteria was achieved. The complete elimination of the biofilm was not obtained, probably due to the single antibacterial administration. A repeated administration may be needed to obtain a complete eradication of the infection, and this represents one of the limitations of the study. However, this is the first study reporting the use of a triple antibiotic paste in the treatment of PI. Nevertheless, the study’s results encourage further research to analyze both potentials and limitations of the protocols.

Another limitation of the present study is inherent to the in vitro design. Real clinical conditions, as well as the individual host responses, could not be perfectly simulated. However, the results obtained suggested that the use of GYPAP in combination with 3MIX could be suggested as a viable protocol for PI, warranting further in vivo investigation.

The efficacy of this new protocol may be related to the combination of a mechanical treatment (GYPAP) which physically reduces the extent of the biofilm, and the antibacterial treatment (3MIX) which kills viable bacteria, thus resulting in an increased biocompatibility. Obtaining a biocompatible surface is mandatory to permit bone regeneration and reosseointegration of the implant, which is the main goal of treating PI [53,54].

## 5. Conclusions

The combination of GYPAP and 3MIX on SLA Ti disks showed the most relevant bactericidal effects capable to achieve a level of biocompatibility sufficient to allow ASC growth.

Within the limitations implicated in the present in vitro study, data showed that the combination of GYPAP and 3MIX could be suggested rather than the other protocols, especially in the presence of rough surfaces such as SLA. This result could have clinical implications in the treatment of PI, which is currently still considered challenging, paving the way toward further in vivo research that is needed to confirm the current results.

## Data Availability

The datasets generated during and/or analyzed during the current study are available from the corresponding author on reasonable request.

## References

[1] Jung R.E., Zembic A., Pjetursson B.E., Zwahlen M., Thoma D.S. (2012). Systematic review of the survival rate and the incidence of biological, technical, and aesthetic complications of single crowns on implants reported in longitudinal studies with a mean follow-up of 5 years. Clin. Oral Implants. Res..

[2] Esposito M., Hirsch J.M., Lekholm U., Thomsen P. (1998). Biological factors contributing to failures of osseointegrated oral implants. (I) Success criteria and epidemiology. Eur. J. Oral Sci..

[3] Troiano G., Lo Russo L., Canullo L., Ciavarella D., Lo Muzio L., Laino L. (2018). Early and late implant failure of submerged versus non-submerged implant healing: A systematic review, meta-analysis and trial sequential analysis. J. Clin. Periodontol..

[4] Salcetti J.M., Moriarty J.D., Cooper L.F., Smith F.W., Collins J.G., Socransky S.S., Offenbacher S. (1997). The clinical, microbial, and host response characteristics of the failing implant. Int. J. Oral Maxillofac. Implants.

[5] Renvert S., Persson G.R., Pirih F.Q., Camargo P.M. (2018). Peri-implant health, peri-implant mucositis, and peri-implantitis: Case definitions and diagnostic considerations. J. Periodontol..

[6] Pesce P., Menini M., Tealdo T., Bevilacqua M., Pera F., Pera P. (2014). Peri-implantitis: A systematic review of recently published papers. Int. J. Prosthodont..

[7] Albrektsson T., Isidor F., Lang N.P., Karring T. (1994). Consensus report: Implant therapy. Proceedings of the 1st European Workshop on Periodontology.

[8] Mombelli A., Lang N.P. (1998). The diagnosis and treatment of peri-implantitis. Periodontol. 2000.

[9] Canullo L., Signorini L., Pistilli R., Patini R., Pistilli V., Pesce P. (2019). A prospective case series on surgical treatment of circumferential and semi-circumferential defects due to peri-implantitis. Braz. Oral. Res..

[10] Mir-Mari J., Mir-Orfila P., Figueiredo R., Valmaseda-Castellon E., Gay-Escoda C. (2012). Prevalence of peri-implant diseases. A cross-sectional study based on a private practice environment. J. Clin. Periodontol..

[11] Atieh M.A., Alsabeeha N.H., Faggion C.M., Duncan W.J. (2013). The frequency of peri-implant diseases: A systematic review and meta-analysis. J. Periodontol..

[12] Subramani K., Wismeijer D. (2012). Decontamination of titanium implant surface and re-osseointegration to treat peri-implantitis: A literature review. Int. J. Oral Maxillofac. Implants.

[13] Mussano F., Rovasio S., Schierano G., Baldi I., Carossa S. (2013). The effect of glycine-powder airflow and hand instrumentation on peri-implant soft tissues: A split-mouth pilot study. Int. J. Prosthodont..

[14] Kotsakis G.A., Olmedo D.G. (2021). Peri-implantitis is not periodontitis: Scientific discoveries shed light on microbiome- biomaterial interactions that may determine disease phenotype. Periodontol. 2000.

[15] Romanos G.E., Gupta B., Yunker M., Romanos E.B., Malmstrom H. (2013). Lasers use in dental implantology. Implant Dent..

[16] Mellado-Valero A., Buitrago-Vera P., Sola-Ruiz M.F., Ferrer-Garcia J.C. (2013). Decontamination of dental implant surface in peri-implantitis treatment: A literature review. Med. Oral. Patol. Oral. Cir. Bucal..

[17] Charalampakis G., Rabe P., Leonhardt A., Dahlen G. (2011). A follow-up study of peri-implantitis cases after treatment. J. Clin. Periodontol..

[18] Persson L.G., Ericsson I., Berglundh T., Lindhe J. (2001). Osseointegration following treatment of peri-implantitis and replacement of implant components. An experimental study in the dog. J. Clin. Periodontol..

[19] Tribst J.P.M., Dal Piva A.M.O., Shibli J.A., Borges A.L.S., Tango R.N. (2017). Influence of implantoplasty on stress distribution of exposed implants at different bone insertion levels. Braz. Oral. Res..

[20] Dhaliwal S.K.S., Knights J., Albuquerque R.F. (2021). Microbial Biofilm Decontamination on Dental Implant Surfaces: A Mini Review. Front. Cell. Infect. Microbiol..

[21] Lo Giudice R., Tribst J.P.M. (2020). Dental Materials Coatings: Effect on the Clinical Behavior. Coatings.

[22] Wennerberg A., Albrektsson T. (2009). Effects of titanium surface topography on bone integration: A systematic review. Clin. Oral Implants Res..

[23] Mussano F., Genova T., Serra F.G., Carossa M., Munaron L., Carossa S. (2018). Nano-Pore Size of Alumina Affects Osteoblastic Response. Int. J. Mol. Sci..

[24] Ayoub H.M., Gregory R.L., Tang Q., Lippert F. (2020). Influence of salivary conditioning and sucrose concentration on biofilm-mediated enamel demineralization. J. Appl. Oral. Sci..

[25] Leonhardt A., Olsson J., Dahlén G. (1995). Bacterial colonization on titanium, hydroxyapatite, and amalgam surfaces in vivo. J. Dent. Res..

[26] Thurnheer T., Belibasakis N. (2016). Incorporation of staphylococci into titanium-grown biofilms: An in vitro “submucosal” biofilm model for peri-implantitis. Clin. Oral. Implants Res..

[27] Roehling S., Astasov-Frauenhoffer M., Hauser-Gerspach I., Braissant O., Woelfler H., Waltimo T., Kniha H., Gahlert M.J. (2017). In Vitro Biofilm Formation on Titanium and Zirconia Implant Surfaces. J. Periodontol..

[28] Canullo L., Genova T., Wang H.L., Carossa S., Mussano F. (2017). Plasma of Argon Increases Cell Attachment and Bacterial Decontamination on Different Implant Surfaces. Int. J. Oral Maxillofac. Implants..

[29] Canullo L., Genova T., Tallarico M., Gautier G., Mussano F., Botticelli D. (2016). Plasma of Argon Affects the Earliest Biological Response of Different Implant Surfaces: An In Vitro Comparative Study. J. Dent. Res..

[30] Mandras N., Alovisi M., Roana J., Crosasso P., Luganini A., Pasqualini D., Genta E., Arpicco S., Banche G., Cuffini A. (2020). Evaluation of the Bactericidal Activity of a Hyaluronic Acid-Vehicled Clarithromycin Antibiotic Mixture by Confocal Laser Scanning Microscopy. Appl. Sci..

[31] Mussano F., Genova T., Corsalini M., Schierano G., Pettini F., Di Venere D., Carossa S. (2017). Cytokine, Chemokine, and Growth Factor Profile Characterization of Undifferentiated and Osteoinduced Human Adipose-Derived Stem Cells. Stem. Cells Int..

[32] Menini M., Delucchi F., Bagnasco F., Pera F., Di Tullio N., Pesce P. (2021). Efficacy of air-polishing devices without removal of implant-supported full-arch prostheses. Int. J. Oral. Implantol..

[33] Grande F., Mochi Zamperoli E., Pozzan M.C., Tesini F., Catapano S. (2021). Qualitative Evaluation of the Effects of Professional Oral Hygiene Instruments on Prosthetic Ceramic Surfaces. Materials.

[34] Schwarz F., Becker K., Renvert S. (2015). Efficacy of air polishing for the non-surgical treatment of peri-implant diseases: A systematic review. J. Clin. Periodontol..

[35] Lee B.S., Shih K.S., Lai C.H., Takeuchi Y., Chen Y.W. (2018). Surface property alterations and osteoblast attachment to contaminated titanium surfaces after different surface treatments: An in vitro study. Clin. Implant Dent. Relat. Res..

[36] Mombelli A. (2002). Microbiology and antimicrobial therapy of peri-implantitis. Periodontol. 2000.

[37] Canullo L., Penarrocha-Oltra D., Covani U., Rossetti P.H. (2015). Microbiologic and clinical findings of implants in healthy condition and with peri-implantitis. Oral Maxillofac. Surg..

[38] Serino G., Ström C. (2009). Peri-implantitis in partially edentulous patients: Association with inadequate plaque control. Clin. Oral. Implants Res..

[39] Pesce P., Canullo L., Grusovin M.G., de Bruyn H., Cosyn J., Pera P. (2015). Systematic review of some prosthetic risk factors for periimplantitis. J. Prosthet. Dent..

[40] Subramani K., Jung R.E., Molenberg A., Hämmerle C.H. (2009). Biofilm on dental implants: A review of the literature. Int. J. Oral. Maxillofac. Implants.

[41] Menini M., Pesce P., Bagnasco F., Carossa M., Mussano F., Pera F. (2019). Evaluation of internal and external hexagon connections in immediately loaded full-arch rehabilitations: A within-person randomised split-mouth controlled trial. Int. J. Oral. Implantol..

[42] Butera A., Gallo S., Pascadopoli M., Luraghi G., Scribante A. (2021). Ozonized Water Administration in Peri-Implant Mucositis Sites: A Randomized Clinical Trial. Appl. Sci..

[43] Mombelli A., Feloutzis A., Brägger U., Lang N.P. (2001). Treatment of peri-implantitis by local delivery of tetracycline. Clinical, microbiological and radiological results. Clin. Oral. Implants Res..

[44] Renvert S., Lessem J., Lindahl C., Svensson M. (2004). Treatment of incipient peri-implant infections using topical minocycline microspheres versus topical chlorhexidine gel as an adjunct to mechanical debridement. J. Int. Acad. Periodontol..

[45] Renvert S., Lessem J., Dahlen G., Lindahl C., Svensson M. (2006). Topical minocycline microspheres versus topical chlorhexidine gel as an adjunct to mechanical debridement of incipient peri-implant infections: A randomized clinical trial. J. Clin. Periodont..

[46] Bassetti M., Schär D., Wicki B., Eick S., Ramseier C.A., Arweiler N.B., Sculean A., Salvi G.E. (2014). Anti-infective therapy of peri-implantitis with adjunctive local drug delivery or photodynamic therapy: 12-month outcomes of a randomized controlled clinical trial. Clin. Oral. Implants Res..

[47] Hoshino E., Kurihara-Ando N., Sato I., Uematsu H., Sato M., Kota K., Iwaku M. (1996). In-vitro antibacterial susceptibility of bacteria taken from infected root dentine to a mixture of ciprofloxacin, metronidazole and minocycline. Int. J. Endod..

[48] Ding R.Y., Cheung G.S.P., Chen J., Yin X.Z., Wang Q.Q., Zhang C.F. (2009). Pulp Revascularization of Immature Teeth with Apical Periodontitis: A Clinical Study. J. Endod..

[49] Amsden G.W. (2001). Advanced-generation macrolides: Tissue-directed antibiotics. Int. J. Antimicrob. Agents.

[50] Cuffini A.M., Tullio V., Mandras N., Roana J., Scalas D., Banche G., Carlone N.A. (2002). Clarithromycin mediated the expression of polymorphonuclear granulocyte response against streptococcus pneumoniae strains with different patterns of susceptibility and resistance to penicillin and clarithromycin. Int. J. Tissue React..

[51] Mandras N., Roana J., Allizond V., Pasqualini D., Crosasso P., Burlando M., Banche G., Denisova T., Berutti E., Cuffini A. (2013). Antibacterial Efficacy and Drug-Induced Tooth Discolouration of Antibiotic Combinations for Endodontic Regenerative Procedures. Int. J. Immunopathol. Pharmacol..

[52] Burrell R.C., Walters J.D. (2008). Distribution of systemic clarithromycin to gingiva. J. Periodontol..

[53] Figuero E., Graziani F., Sanz I., Herrera D., Sanz M. (2014). Management of peri-implant mucositis and peri-implantitis. Periodontol. 2000.

[54] Canullo L., Schlee M., Wagner W., Covani U., Montegrotto Group for the Study of Peri-implant Disease (2015). International Brainstorming Meeting on Etiologic and Risk Factors of Peri-implantitis, Montegrotto (Padua, Italy), August 2014. Int. J. Oral Maxillofac. Implants..

